# Coplanar UHF RFID tag antenna with U-shaped inductively coupled feed for metallic applications

**DOI:** 10.1371/journal.pone.0178388

**Published:** 2017-06-01

**Authors:** Karrar Naji Salman, Alyani Ismail, Raja Syamsul Azmir Raja Abdullah, Tale Saeedi

**Affiliations:** Department of Computer and Communication Systems, Faculty of Engineering, Universiti Putra Malaysia, 43400 UPM Serdang, Selangor, Malaysia; Lanzhou University of Technology, CHINA

## Abstract

In this paper, we present a novel compact, coplanar, tag antenna design for metallic objects. Electrically small antenna has designed for a UHF RFID (860–960 MHz) based on a proximity-coupled feed through. Furthermore, two symmetrical Via-loaded coplanar grounds fed by a U-shaped inductively coupled feed through an embedded transmission line. This configuration results in an antenna with dimensions of 31 × 19.5 × 3.065 mm^3^ at 915 MHz, and the total gain for the antenna is 0.12 dBi. The Via-loaded coplanar and U-shaped inductively coupled feeds allow the antenna to provide flexible tuning in terms of antenna impedance. In addition, a figure of merit is applied for the proposed tag antenna, and the results are presented. The read range is measured to be 4.2 m, which is very close to simulated values. This antenna measurement shows very good agreement with simulations.

## Introduction

RADIO frequency identification has received considerable attention in recent years because of the provided long read range and low manufacturing cost. The gain of the antenna, the efficiency, the chip power consumption [1], and the microchip sensitivity power play a main role in providing the long read range. The performance of an antenna depends on the overall size, reading distance, and tagging object’s compatibility with the antenna [2]. In general, the main goal for the ultra-high frequency (UHF) RFID tag antenna design is to decrease its size, expand its bandwidth, and enhance its gain, whereas enough budgets should be reserved for the reliability and robustness of the system.

Moreover, the following issues concerning RFID applications are raised: when a tag is attached directly to the tagged objects, the incident electromagnetic wave will reflect back with a reverse phase pattern from a metallic surface. Accordingly, this will alter the antenna’s radiation pattern, resonant frequency, and input impedance. The change is a function of the material and the distance from it and dimensions of the object [3], [4]. The antenna efficiency degradation is the major obstacle to the widespread introduction of RFID technology in the UHF band. This degradation results in a significant change in antenna efficiency, which is caused by the nearby object, especially for objects composed of metallic materials [5], [6].

Lately, demands have been observed for enhancing the gain in RFID tag antenna design. The gain enhancement can be obtained through many routes. Metamaterials represent one such route; however, these materials are very complex and quite costly to design [7]. Reducing the multiresonant modes into one mode could be another choice for enhancing the gain. Another option is to use 3-D antennas [8]; however, meander-line antennas are large and tricky to manufacture, especially considering the size and cost are of utmost importance in RFID applications. It is best to try to make use of the available space as much as possible for the designed antenna because the electrical size of the antenna is proportional to the achievable radiation efficiency. In other words, the longer the electrical size of the antenna, the better the achievable radiation efficiency will be [9]. Furthermore, antennas with high permittivity and low-loss substrates are used for electrically small antennas; however, they suffer from narrow bandwidths and high manufacturing costs [10], [11].

Similarly, U-shaped parasitic elements have been utilized to obtain good inductive coupling [12]; however, the process is quite complicated because of the addition of a matching network, and the bandwidth is limited.

A good method for reducing the size of the antenna is using a loaded via-patch [13]; this method not only changes the current flow but also affects the radiation efficiency. Normally, the load patch feed for this type of antenna can use the method of proximity-coupled feed [14]; this method usually leads to a minimum antenna size λ_0_/8, whereas the volume of the antenna remains high [15]. Moreover, the loaded via-patches, known as a capacitive feed, provide the benefits of enormous bandwidth and small volume. Radiation cancellation is predominant in the current feed method.

In [16], a comparison is introduced between a coplanar patch antenna and a microstrip patch antenna specifically in terms of efficiency and bandwidth. The results showed that the coplanar antenna obtains a higher efficiency and increased bandwidth. Furthermore, some coplanar antennas introduced by [17], [18] can be used for RFID applications. However, uncertainties in the use of the antennas exist because the studies did not perform RFID specification measurements; rather, they used 50 Ω conventional measurements.

Finally, the coupled inductive feeding was first introduced by [19]. Later, different types of inductively coupled feeds, such as in [20], [21], [22], [23], [24], were introduced. Recently, U-shaped inductively coupled feeds were used to obtain gain enhancements in [25].

This paper is devoted to designing ultra-high-frequency RFID tag antennas with gain-improved characteristic for metallic applications. Motivated by coplanar technology, U-shaped inductively coupled feeds, and HIS-conventional techniques, a loaded via-patch construction is developed.

Today, there is increasing demand for low-cost tag antennas, the same as for antenna-based sensors [26] (such as in structural health monitoring [27]), where the shifting in the [resonant] frequency represents a great choice for universal sensing applications [28]). Thus, an antenna with high-efficiency radiation characteristics is more beneficial than narrow bandwidths [29]for our applications. Generally, a compromise exists between bandwidth and efficiency for a given antenna volume [30].

We introduce a dual-layer coplanar transmission line tag antenna and a U-shaped feed added to the middle of the top layer as a feed network to provide the inductive effect for the antenna. By specially designing the antenna configuration, we correctly remove the current cancellation and hence increase the radiation for the proposed tag antenna. It is convenient to note that the tempting features and good characteristics were the only motivation to utilize a coplanar type of antenna as a replacement method for conventional types of tag antennas [31, 32], [33], [34], [35], especially considering that the growing needs for increasing gain, expanding bandwidth and reducing the size are the most crucial demands in good antenna designs. We structure our paper as follows. First, we propose a loaded via-coplanar ground, multi-layer antenna design based on a coupled proximity feed. Second, we introduce U-shaped inductively coupled feeding around the coplanar transmission line. Next, we examine impedance matching, and the radiation performance is analyzed with this proposed antenna for metallic objects. Finally, the performances of the proposed electrically small antenna (ESA) are assessed by applying a figure of merit (FoM).

## Antenna structure

**Fig 1:**
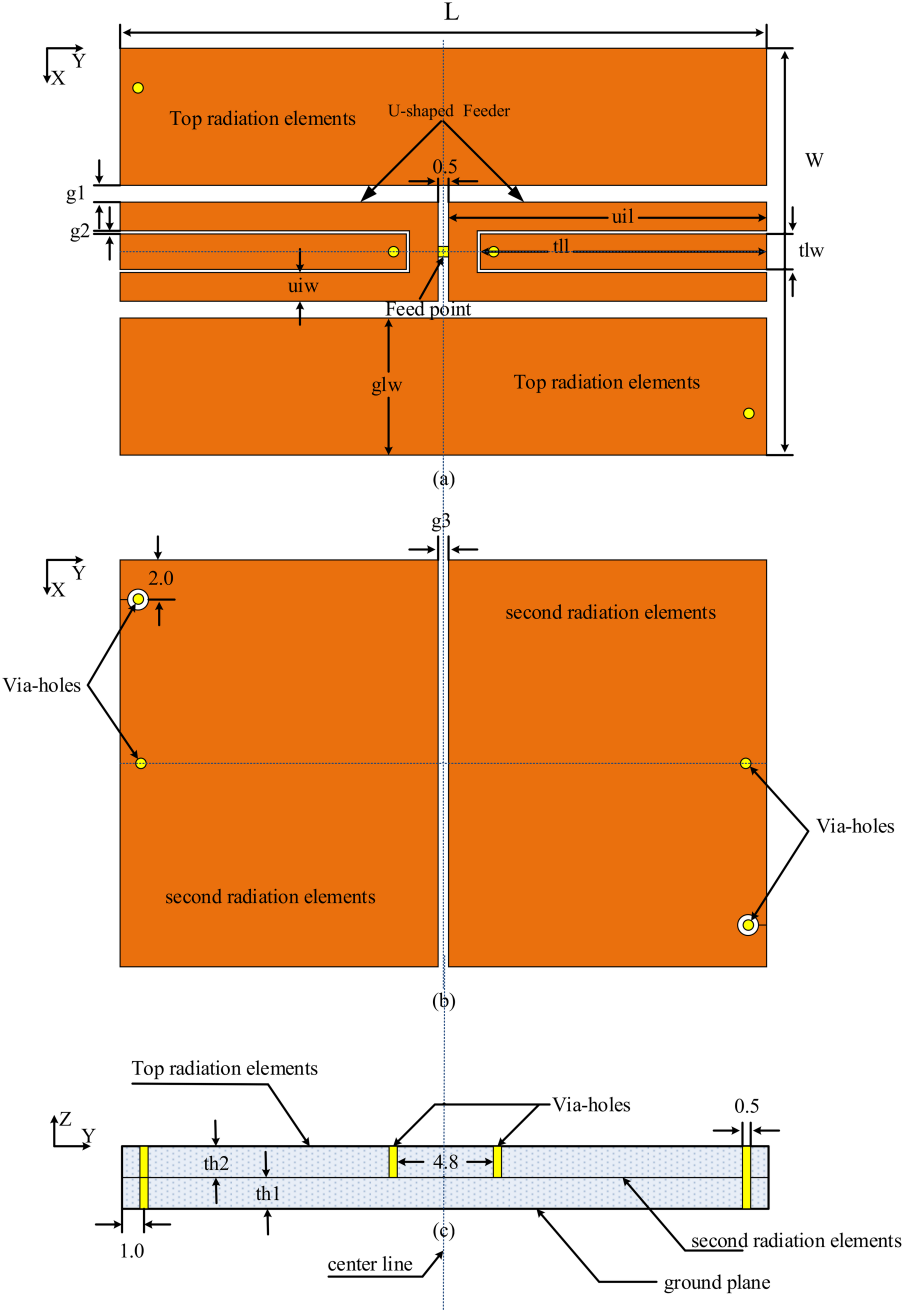
Structural configuration of the proposed antenna: (a) top radiation elements, (b) second radiation elements, and (c) side view.

The structure of the proposed antenna is composed of two layers. The top layer substrate is polytetrafluorethylene (PTFE), with a thickness of 1.5 mm, a dielectric loss tangent of 0.001, and a relative permittivity of 2.55, whereas the bottom layer is FR4. The FR4 thickness is 1.53 mm, the dielectric loss tangent is 0.025, and the relative permittivity is 4.3. The overall size of the antenna is 31 × 19.5 × 3.065 mm^3^. The first layer consists of a loaded via coplanar ground antenna on the top side of the PTFE layer. The two coplanar grounds shorted to the ground plane were printed on the back side. A coplanar transmission line is introduced in the center of the gap. The U-shaped inductive line is introduced around the transmission line as a couple feeder. Shown in Fig 1(a)–1(c), the loaded via coplanar ground is referred to as the top-layer radiation elements, which can be observed in Fig 1(c). We design the U-shaped feed point at the top layer for convenient fabrication. Next, the coplanar compact tag antenna loaded via coplanar grounds and inductive feeder were structured. Higher loss FR4 and lower loss PTFE substrates are chosen in this antenna to provide a tradeoff between the bandwidth and efficiency of the antenna. Considering the difficulty in satisfying the coplanar specifications and obtaining a good matching because of the compact shape of the coplanar grounds, an attempt to use U-shaped feeder to overcome possible problems is worth attempting to keep up with the coplanar feature. Furthermore, two more issues inspired us to design a fixable inductive feed. First, in contrast to the 50 Ω conventional antennas, the RFID antenna has a complex impedance that should be matched to a conjugated chip impedance. RFID chips do not have a consolidated slander. Different chips have different impedances. An RFID tag antenna that can match all types of chips may be impossible to design.

Second, all RFID tag antennas are of a single-use design because the information in the chip is exclusive to a certain product. If the RFID chip is standardized to the tag antenna, the tag antenna can be used only once [24]. Based on this, a U-shaped inductively coupled feeder was introduced to overcome the possible conjugate matching problem when a different chip will be utilized for the same designed antenna for a specific application. In other words, the U-shaped inductively coupled feeder can provide a very flexible method to reach a good conjugate matching to our designed antenna if the RFID chip changes to another one that is holding different information for diverse applications.

The substrate selection encompasses choosing materials with low loss, low cost and low permittivity characteristics. This means that high loss (low cost) materials are not favorable. A coplanar tag design substrate was chosen to obtain these characteristics, and specifically, the focus should be on choosing the dielectric constant of the substrate to be as low as possible, thereby obtaining the best performance, as indicated in [30]. Furthermore, the widespread adoption of UHF RFID technology necessitates a low cost.

## Antenna design

We chose a coplanar tag antenna because of its low dispersion, good efficiency, unlimited size reduction ability [36] and, most importantly, increased gain for lossy types of antennas, which can be obtained by increasing the radiation efficiency. The feed network chosen here is a U-shaped inductively coupled network near the transmission line and the two coplanar grounds. Impedance tuning for this type of electrical small antenna is difficult to obtain.

Accordingly, an inductive feeder is proposed to solve this problem. Furthermore, some radiation is produced near the small gap in the middle [37], thus our reason for calling it the second radiator layer. Simultaneously, we utilize a U-shaped inductively coupled feeder with two loaded via coplanar grounds to act as the first radiator because, in designs for obtaining high efficiency, compact antenna slotting is not a valid solution [38]. The coplanar ground plans are fed by a U-shaped feeder in addition to the proximity-feed coupled method through the network underneath. The topology choice utilized in the coplanar design is meant to ameliorate the space usage as much as possible. Usually, conventional mounted metal tag antennas suffer from the disadvantage of poor performance because of the critical limitation of their profile.

The tag range can vary up to 40% with 3-dB variations in the impedance matching or microchip sensitivity [39]. Thus, the matching between the chip and antenna is especially significant. Moreover, a tag antenna has to be designed to satisfy the band, which is specified on a per-region basis (920–925 MHz in China, 916–924 MHz in Japan, 866–869 MHz in Europe, and 902–928 MHz in the U.S.A.). Thus, a shift in the operating frequency or variations in impedance caused by manufacturing error or the tagged objects can be efficiently addressed by increasing the bandwidth [40].

**Fig 2:**
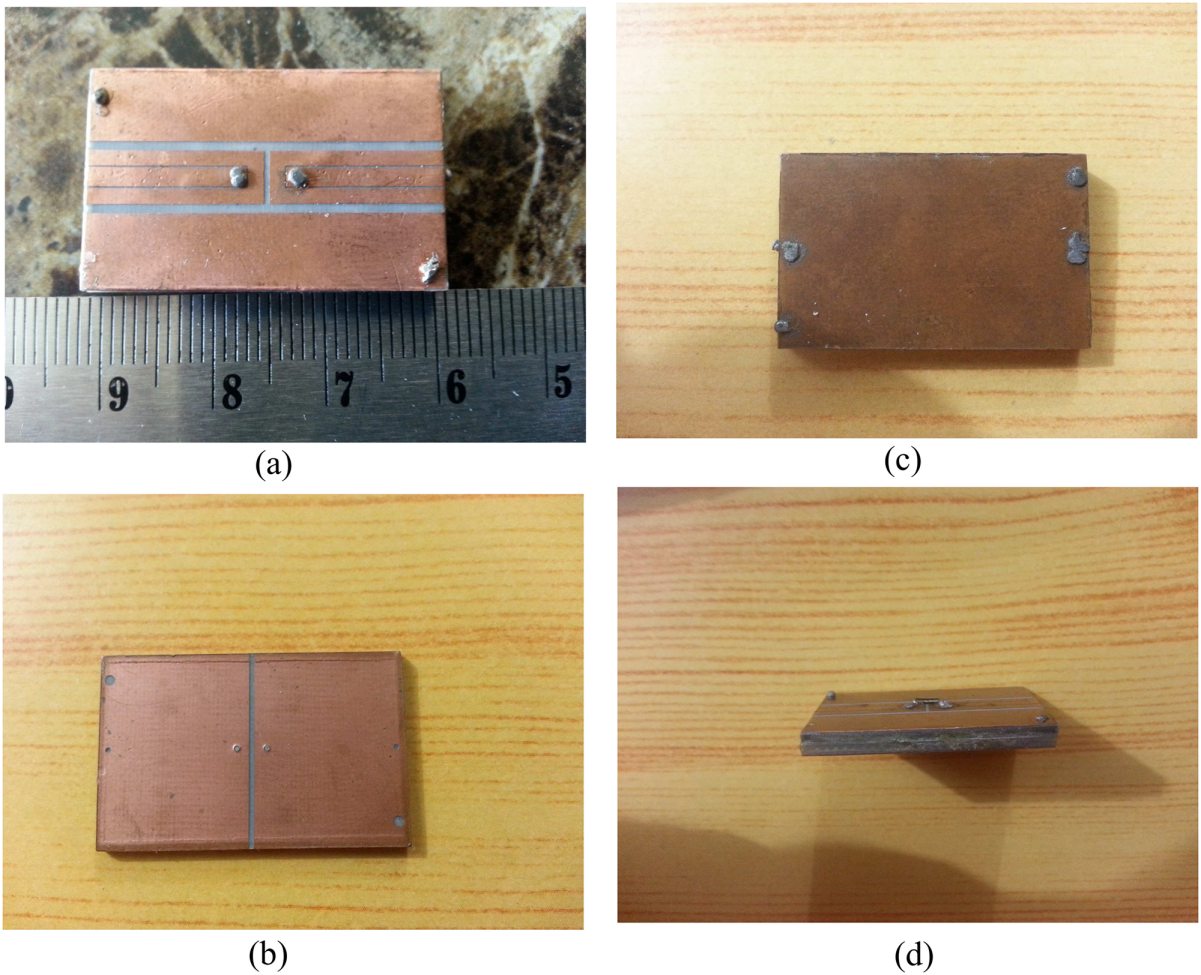
Fabricated antenna: (a) top radiation elements, (b) second radiation elements, (c) back elements and (d) side view. With L = 31 mm, W = 19.5mm, glw = 6.57 mm, g1 = 0.805 mm, g2 = 0.15 mm, tlw = 1.7 mm, uiw = 1.375 mm, uil = 15.25 mm, tll = 13.725 mm, and g3 = 0.5 mm.

This antenna was simulated and modeled using Computer Simulation Technology (CST) (Microwave Studio and Design Studio). This prototype antenna is devised for conjugate impedance matching for the MURATA magicstrap LXMS31ACNA-011 chip when the input impedance at 915 MHz [41], and the formal reading sensitivity is −8 dBm (160 *μ*W). The input impedance for this chip is *Z*_*ic*_ = 25 − *j*200 Ω at 915 MHz. To improve power delivery, the input impedance for the antenna should be close to the conjugate for *Z*_*ic*_ for the chip. For metallic applications [42], the validations were conducted by modeling the tag antenna on a 200 × 200 mm^2^ prefect electrical conductor (PEC). The antenna was optimized for a metallic feed and fabricated using an etching method, as shown in Fig 2.

The maximum read range (*d*_*max*_) can be obtained according to the propagation hypothesis based on the line of sight occurring between the tag and the reader. The equation is given by [43].

$$\begin{array}{c} d_{max}\left(\emptyset ,\vartheta \right)=\frac{\lambda }{4\pi }\sqrt{\frac{P_{t}G_{t}}{Pth}}\;\rho \tau \;G_{tag}\left(\emptyset ,\varphi \right) \end{array}$$ (1)

where λ is the wavelength, *P*_*t*_ is the transmitted power of the reader, *ρ* is the polarization mismatch between the tag and the reader, *P*_*th*_ is the power minimum threshold to activate the tag, *τ* is the coefficient of the transmission power, *G*_*t*_ is the gain of the transmitting antenna, and *G*_*tag*_ is the gain of the tag. Regarding impedance matching between the antenna and the chip, *τ* is expressed as the impedance mismatch that can occur between the chip and the antenna, (*Z*_*c*_ = *R*_*c*_ + *X*_*c*_) for the chip, and (*Z*_*a*_ = *R*_*a*_ + *X*_*a*_) for the antenna. This impedance mismatching can be written as

$$\begin{array}{c} \tau =1-|S_{11}{|}^{2}=1-|\frac{Zc-Za^{*}}{Zc+Za}{|}^{2}=\frac{4Rc\;Ra}{|Zc+Za{|}^{2}}\leq 1 \end{array}$$ (2)

where Za* is the conjugate impedance value of the tag antenna.

## Parametric study effect

**Fig 3:**
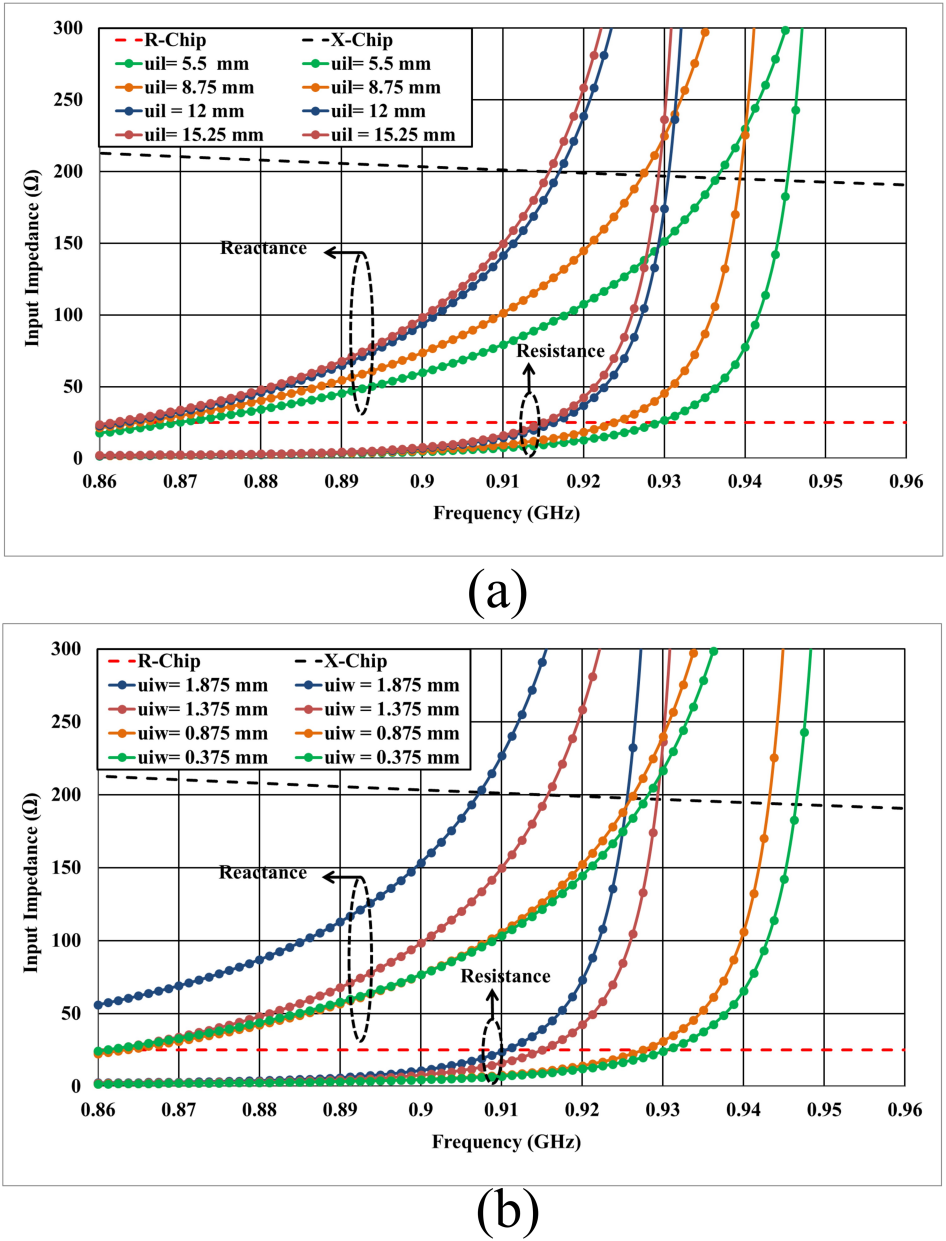
Input impedance for different values of: (a) the U-shaped inductive coupled feeder length (uil), and (b) the U-shaped inductive coupled feeder width (uiw) mounted on a metallic plate with dimensions of 200 × 200 mm^2^, where R-Chip and X-Chip represent the real and imaginary parts of the conjugate chip impedance.

Given the significant effects of all the antenna parameters on the antenna performance, especially for the ESA parameters [21], the U-shaped feeder structure plays a main role for antenna optimal conjugate matching performance as in Eq (5). The feeder configuration has been introduced as shown in Fig 1(a). The use of the U-shaped inductive feed mainly attempts to make the tag antenna very flexible in terms of tuning parameters. As is known, the coplanar transmission line has specific parameters that characterize it, and a U-shaped feeder is the best solution for providing the tag antenna with an easy tuning ability beyond the coplanar changing specifications. The feeder length (uil) and width (uiw) significantly control the strength of the coupling as illustrated in Fig 3.

**Fig 4:**
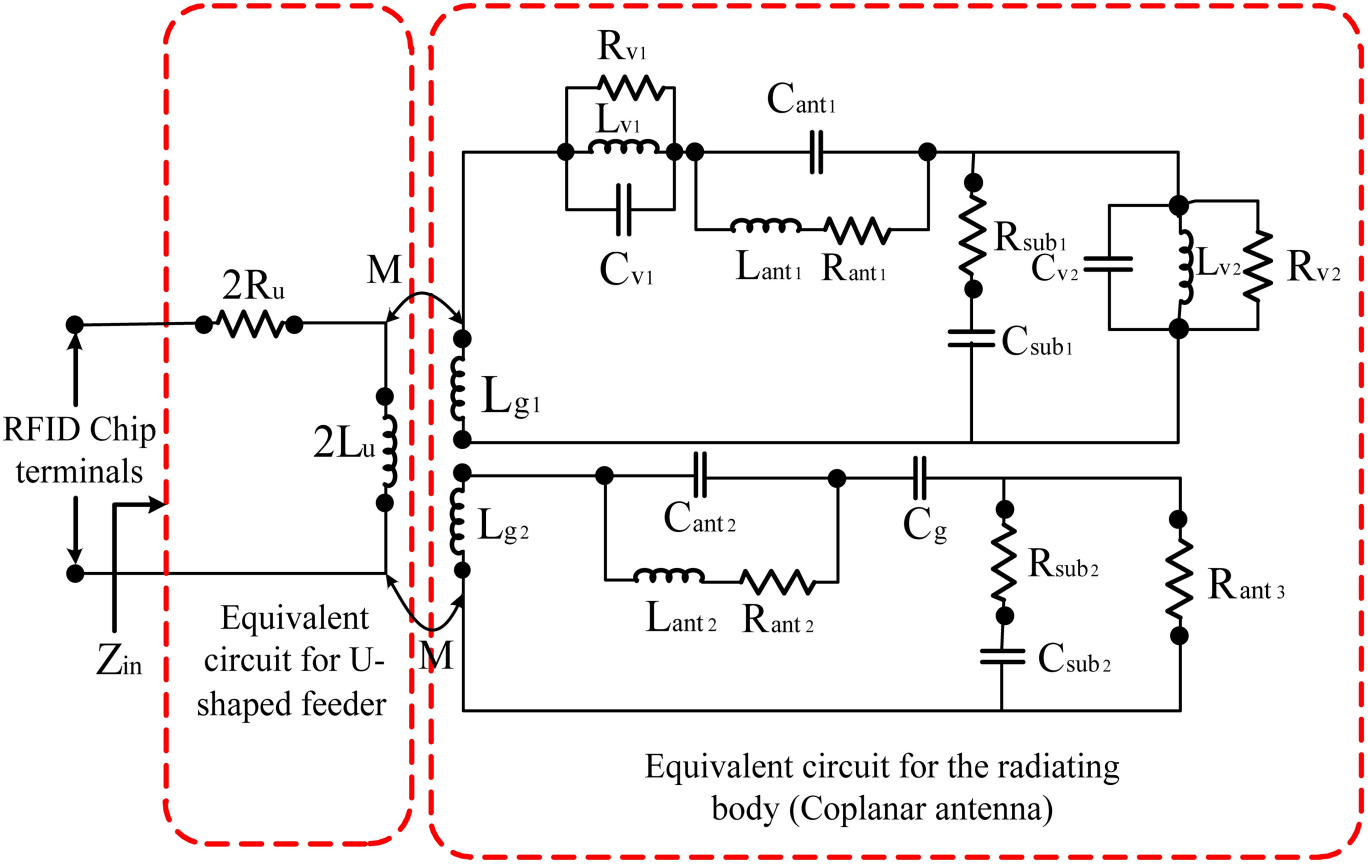
Equivalent circuit model for U-shaped inductively coupled feed.

The equivalent circuit model for the U-shaped inductively coupling feed structure with the tag antenna radiating body is shown in Fig 4.

The inductive coupling is also modeled as a transformer. The input impedance for antenna *Z*_*ant*_ is reported by [19].

$$\begin{array}{c} Z_{in}=R_{in}+jX_{in}=Z_{ufeed}+\frac{{\left(2\pi f_{M}\right)}^{2}}{Z_{ant}} \end{array}$$ (3)

where *Z*_*ant*_ is the impedance of the radiating coplanar antenna, *Z*_(*Ufeed*)_ is the impedance of the U-shaped feeds, and *M* is the mutual inductance between them. We assume that the radiating coplanar body is infinitely long. The input impedance for the U-shaped feeds can be written as [25].

$$\begin{array}{c} Z_{ufeed}=2R_{u}+j2\pi f\left(2L_{u}\right) \end{array}$$ (4)

where *L*_*U*_ is the self-inductance of the U-shaped feed. When *f* = *f*_*o*_, assuming that the substrate effect is minimal, the input impedance for the resonant frequency of the radiating coplanar body can be written as follows [25]:

$$\begin{array}{c} Z_{in}=\left[2R_{u}+\frac{{\left(4\pi f_{o}M\right)}^{2}}{3R_{ant}}\right]+j4\pi f_{o}L_{u} \end{array}$$ (5)

The input reactance is noticeable.

**Fig 5:**
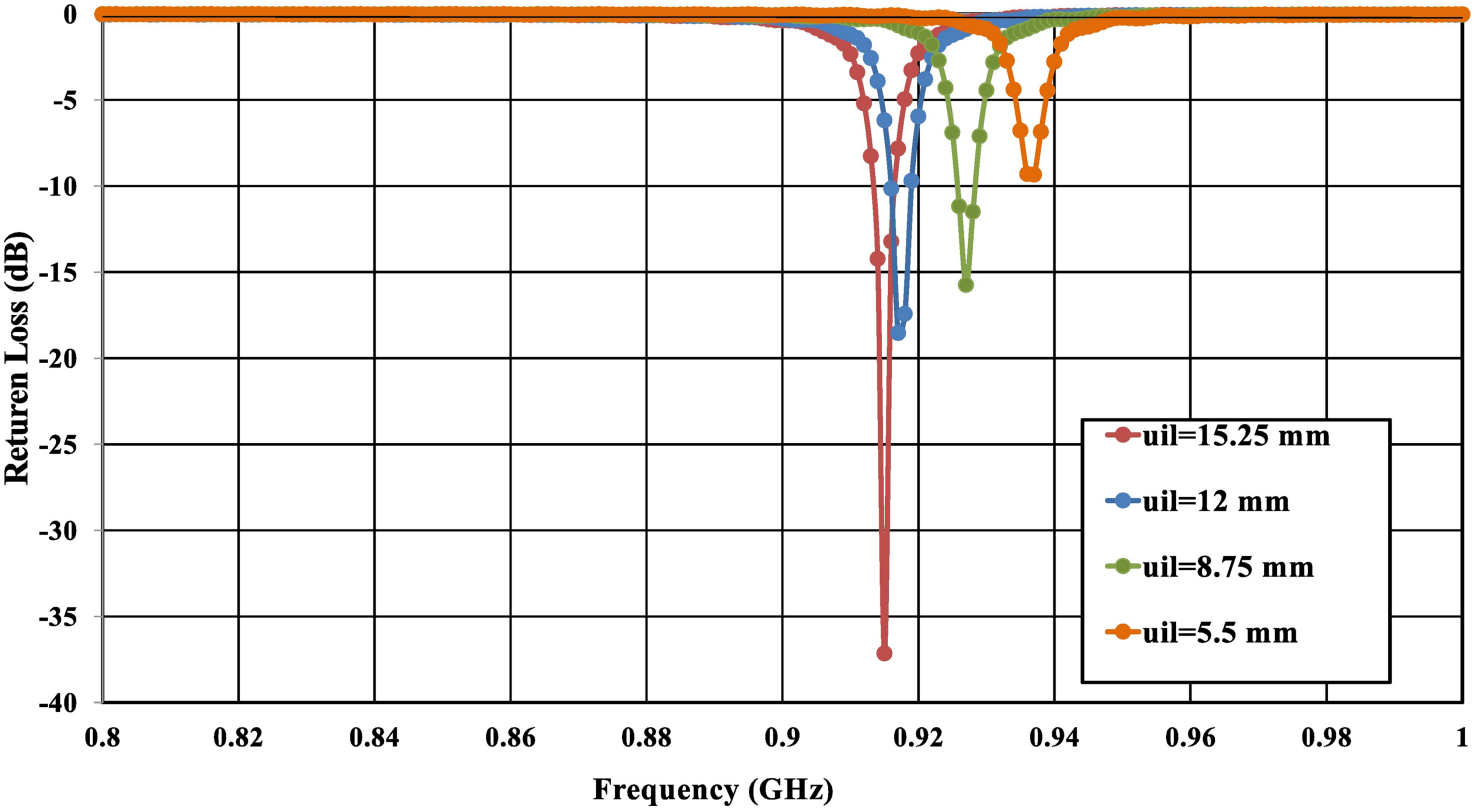
Simulated results of return loss (*S*_11_) for the proposed coplanar tag antenna mounted on a metal sheet with dimensions of 200 × 200 m^2^, with a variation of U-shaped feeder length (uil).

From Eq (5), it can be noted that the input reactance depends only on the length of the U-shaped feeder (uil); however, the input resistance can be adjusted by (*M*) and *R*_*ant*_ regardless of the coplanar body of the tag antenna at a specific resonant frequency. The length of the U-shaped feeder is primarily determined by the reactance part of the antenna, as shown in Fig 3(a). By decreasing the length of the feeder, the resonant frequency will shift to higher frequencies due to a reduction in the mutual coupling as illustrated in Fig 5. Furthermore, The simulated results illustrated that any decrement of the U-shaped feed length (uil) shifts up the tag antenna resonance frequency because the strength of the inductive coupling is controlled by the mutual coupling between the U-shaped feeder and antenna body. The dimensions of U-shaped feeder are very crucial to determine a perfect matching between the chip and the tag antenna [25], as demonstrated in Fig 5.

**Fig 6:**
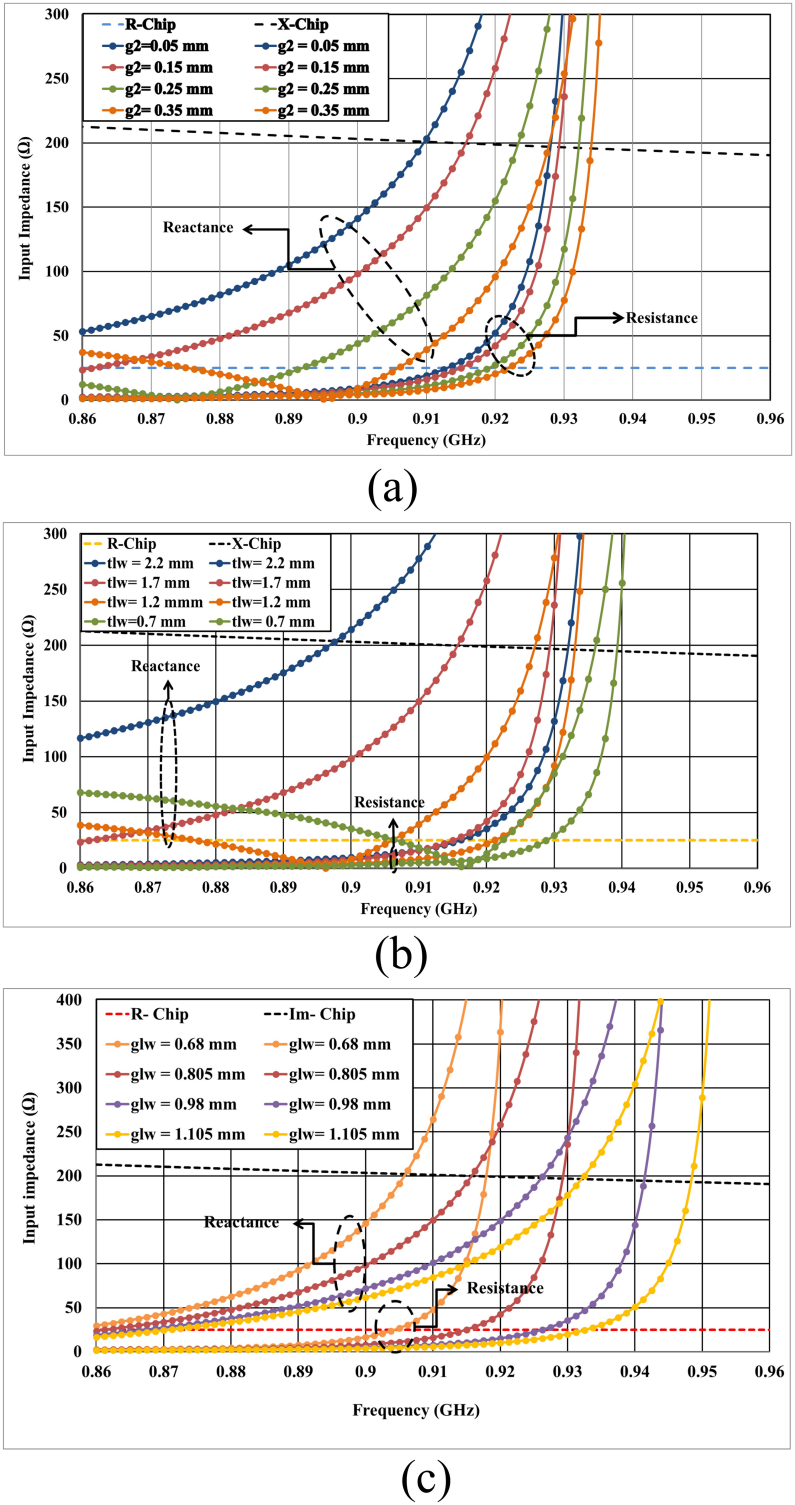
Input impedances of the proposed coplanar tag antenna mounted on a metal sheet with dimensions of 200 × 200 m^2^, with a variation of: (a) g2, (b) tlw, and (c) glw, where R-Chip and X-Chip (or Im-Chip) represent the real and imaginary parts of the conjugate chip impedance.

The coupling effect of the transmission line can be significantly controlled by the gap between the feeder and transmission line tag antenna (g2), where the center frequency could be shifted toward higher frequencies using an increased gap (g2) due to the decreasing of the coupling effect on the transmission line and the tag coplanar antenna. The larger the gap is, the weaker the coupling will be, which will negatively affect the resonant frequency. However, the effect of increasing the gap (g2) can be removed by increasing the length of the antenna due to the increment of its inductance, which is not a favorable solution [44]. Similarly, the transmission line width (tlw) has the same effect as the gap (g2) and the width of coplanar ground plane (glw) in terms of lowering the coupling effect and shifting the frequency to higher bands as shown in Fig 6.

The width of the U-shaped feeder has a very crucial effect on the tag design as well. The width of our feeder is designed to obtain the optimum performance in the design of the tag. From Fig 3(b), we can see that decreasing the width of the feeder will decrease the inductive effect and thus shift the center frequency toward higher bands. It is worth mentioning that the resonant frequency for our design shifts up as the width of the U-shaped feeder decreases due to the low inductive coupling effect occurring at the feeder.

**Fig 7:**
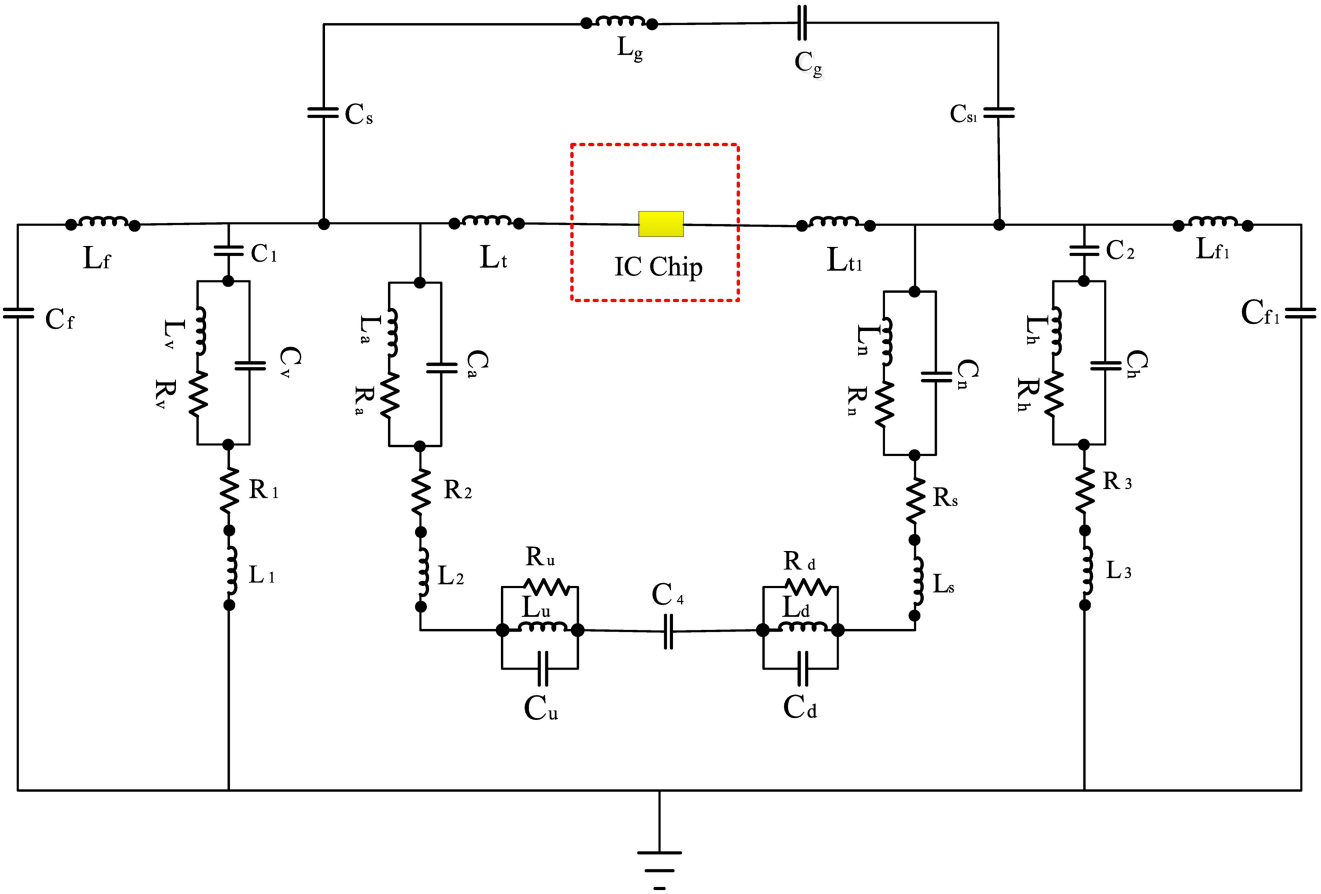
Equivalent circuit model of the proposed RFID tag antenna. Here, *C*_*f*_ = 4 pF, *L*_*f*_ = 39 nH, *C*_1_ = 1 pF, *R*_*v*_ = 1 kOhm, *C*_*v*_ = 1.7 pF, *L*_*v*_ = 50 nH, *R*_1_ = 50 Ohm, *L*_1_ = 1 nH, *C*_*s*_ = 0.56 pF, *L*_*g*_ = 20 nH, *C*_*g*_ = 4.98 pF, *L*_*t*1_ = 100 nH, *R*_*a*_ = 50 kOhm, *C*_*a*_ = 7.92 pF, *L*_*a*_ = 1 nH, *R*_2_ = 50 Ohm, *L*_2_ = 50 nH, *R*_*u*_ = 50 Ohm, *C*_*u*_ = 1 pF, *L*_*u*_ = 1 nH, *C*_4_ = 1 pF, *C*_*s*1_ = 4 pF, *L*_*t*_ = 9.095 nH, *R*_*n*_ = 1 kOhm, *C*_*n*_ = 4 pF, *L*_*n*_ = 50 nH, *R*_*s*_ = 50 Ohm, *L*_*s*_ = 50 nH, *R*_*d*_ = 50 ohm, *C*_*d*_ = 1 pF, *L*_*d*_ = 1 nH, *C*_5_ = 1 pF, *R*_*h*_ = 50 kOhm, *C*_*h*_ = 6.5 pF, *L*_*h*_ = 50 nH, *R*_3_ = 50 Ohm, *C*_3_ = 1 nH, *L*_*f*1_ = 13.575 nH, and *C*_*f*1_ = 5.4 pF.

Finally, the equivalent circuit model for a coplanar tag antenna radiating body, which supplies reasonable insight into how this coplanar tag antenna operates, is shown in Fig 7.

In the equivalent circuit shown in Fig 7, the chip point can be the start based on the combination of inductors and capacitors both in series and in parallel. For the equivalent circuit, we included an inductor for the lines and a capacitor for the gaps. The capacitors, which have been located on both sides of the circuit, are positioned for the fringing fields. Moreover, for the via-hole and the substrate, their exact circuit is obtained [45].

## Antenna measurements

### Impedance matching measurement

**Fig 8:**
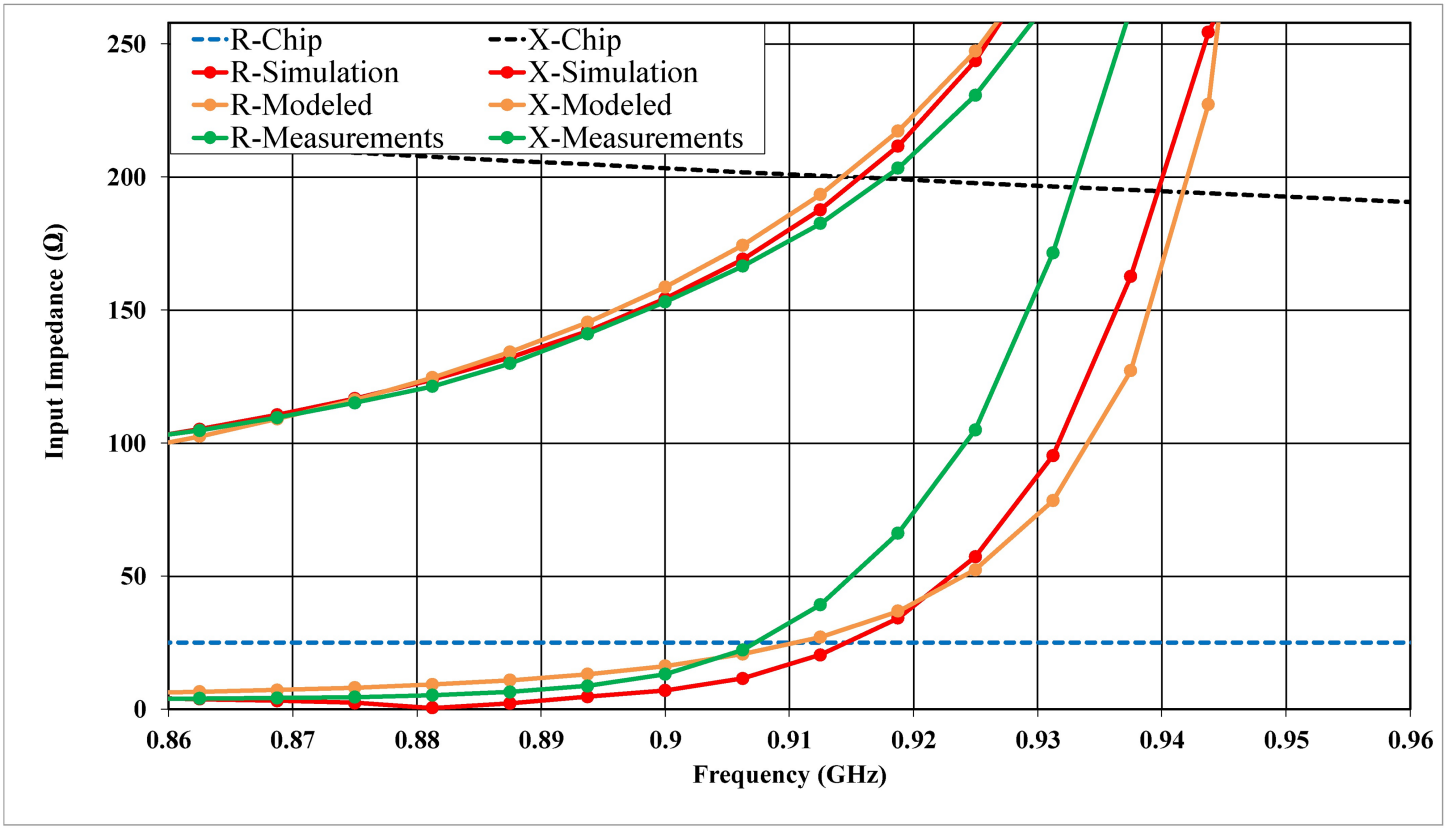
Input impedance simulation and measurement of the proposed tag antenna mounted on a 200 × 200 mm^2^ metallic plane, where R-Chip and X-Chip represent the real and imaginary parts of the conjugate chip impedance.

**Fig 9:**
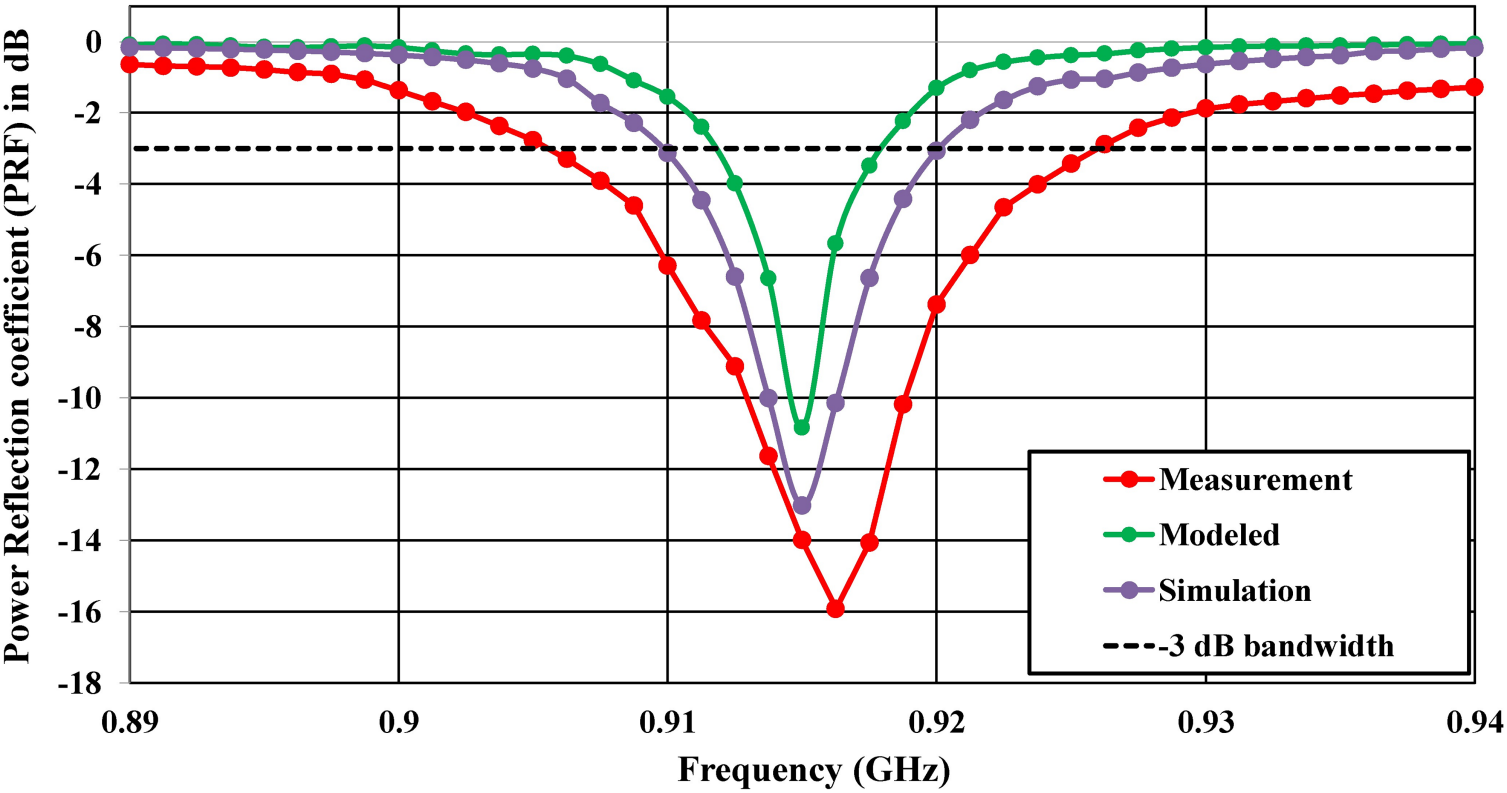
Power reflection coefficient of *S*_11_ (PRC).

**Fig 10:**
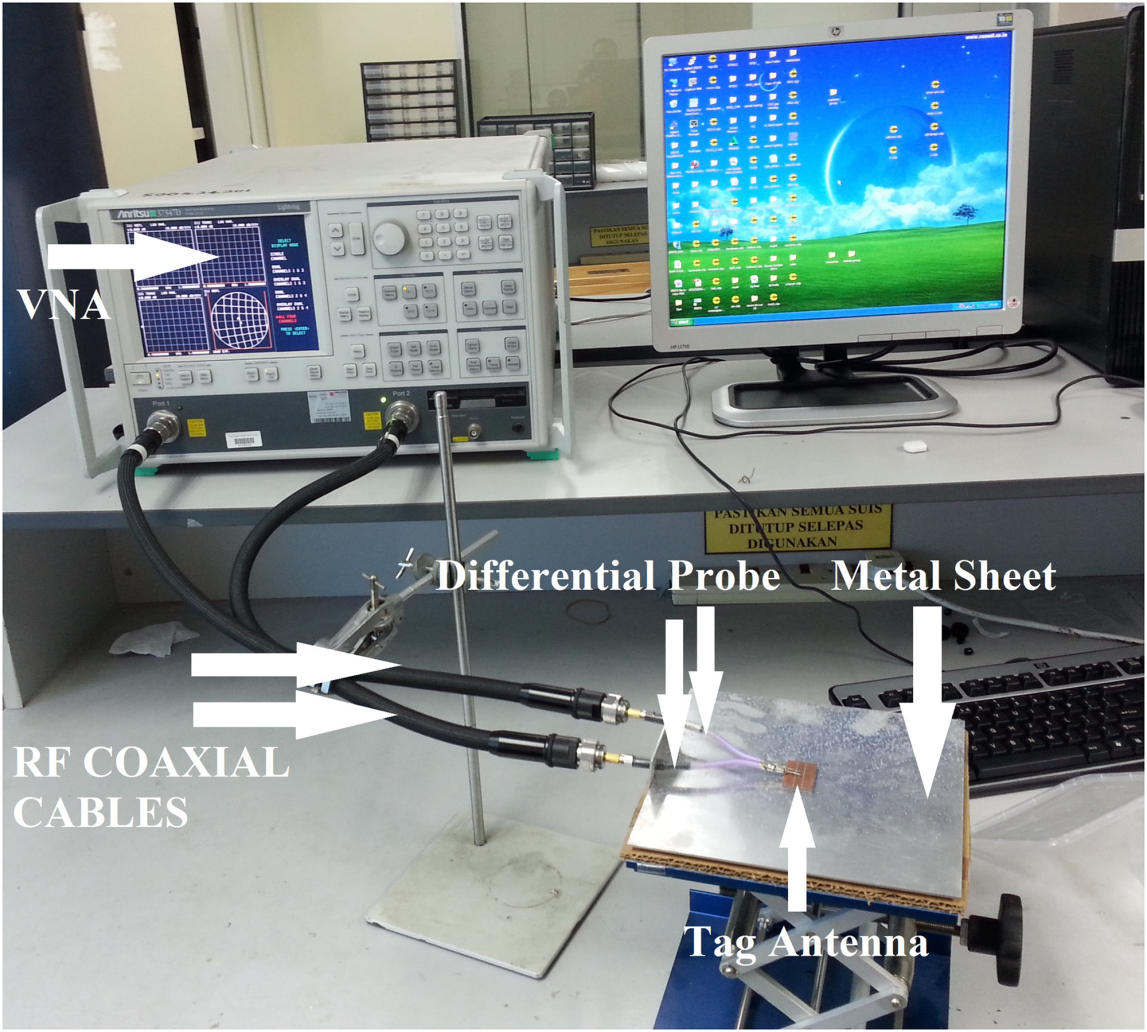
Power reflection coefficient (PRC) of return loss *S*_11_ measurements.

The proposed antenna was verified, and the antenna impedance was obtained using the Anritsu (37347D) vector network analyzer (VNA) in addition to the port-extension technique introduced in [29]. The tag microchip incorporates an energy-storage stage, thereby explaining the strongly capacitive behavior of the input reactance [43]. Thus, the impedance characteristic includes both a real part and an imaginary part, varying around the conjugate of the microchip impedance value at 915 MHz. Fig 8 shows the proposed antenna impedance; moreover, the reflection coefficient of *S*_11_ is calculated in Fig 9. Emulation of a typical environment was the reason that the power reflection coefficient (PRC) of the *S*_11_ measurement was obtained in an ordinary room, as shown in Fig 10. The proposed antenna half-power bandwidth was measured to be 20 MHz (906–926 MHz).

Measuring the impedance is a difficult task for nearly all differential-feed ESAs because various errors can arise when applying the measurement system. Furthermore, the difference between the simulation and measurement results, especially for most differential feeds, should be carefully noted and may be noticed particularly in electrically small antennas such as those in [11], [46], [47], and in the differential-feed asymmetric antennas [48] because they are more likely to exhibit the variation in parameter issue. In our proposed antenna, there are gaps at the top and in-between the two layers, which may lead to a change in the results and shift the frequency to higher frequencies because of the soldering procedure.

In the proposed configuration, the impedance can be tuned by changing the length and the width of the feeder, the resonant frequency will shift to higher frequencies due to a reduction in the mutual coupling, as shown in Fig 3. Although via-holes affect the real part, many factors can be chosen to act as a tuning part for matching impedance. However, the length and the width of the feeder was chosen to be the only parameter for the top layer. By varying these two parameters, we obtain our resonant frequency and accordingly adjust the impedance matching. The accuracy of the measurements will be significantly affected by the imperfections in the test fixture (differential probe) such as the unpreventable abruption between the fixture (differential probe) and the antenna [49], radiation disturbances caused by the conductive shielding layer [50], or poor calibration [51]. A further simulation demonstrates that the accuracy of the impedance measurement is significantly influenced by the dimensions of the test fixture (differential probe).

### 0.1 Realized gain and read range measurements

**Fig 11:**
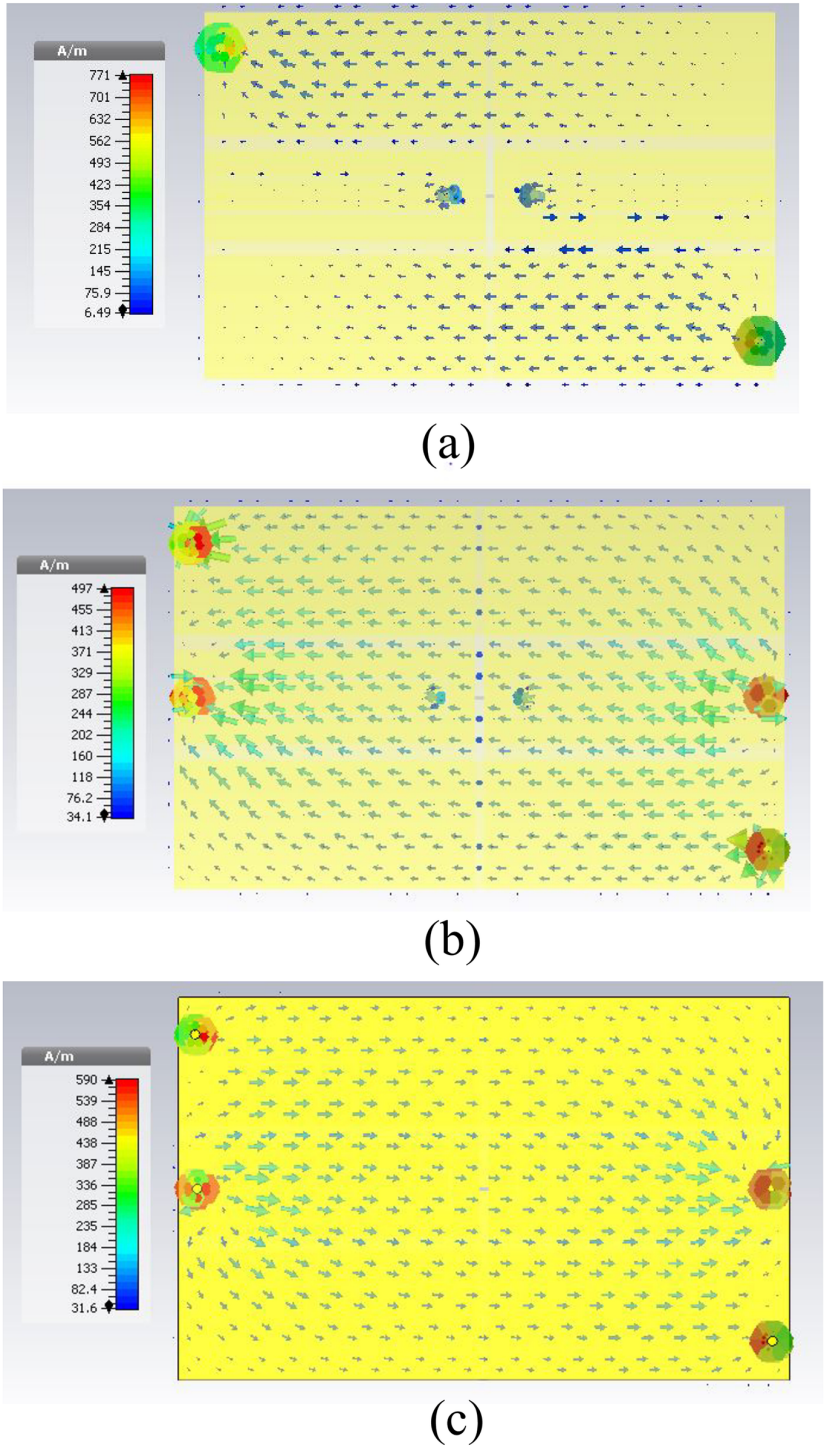
Current distributions of the proposed RFID tag antenna on a metallic ground plane with dimensions of 200 mm × 200 mm: (a) top layer, (b) second layer, and (c) ground plane.

The antenna conductivity is directly related to the antenna current distribution. The simulated antenna current distributions at 915 MHz are shown in Fig 11. The good distribution of via-holes strongly affects achieving a magisterial horizontal current distribution to the radiator layer [52]. Meantime, the current flux in the top radiator layer is mostly influenced by the U-shape inductive feeder, transmission line, and coplanar ground planes through the proximity-coupled method. Consequently, the top layer current magnitude can be seen to be much greater than in the feed layer. Moreover, with the coplanar ground planes, the method helps to increase the electrical length for the proposed antenna based on a convenient current distribution through via-holes. The antenna’s rotationally symmetrical configuration helps to determine the direction of the current flow either in the (negative Y) or (negative X) direction, which can be noticed in Fig 11. Remarkably, the efficiency increased significantly compared with the conventional meander line and PIFAs or fractal antennas [38], [53] because of the increase in radiation at all edges in the antenna. Furthermore, it is worth mentioning that, by encouraging a slight difference in the somatic length of the designed via-coplanar grounds, the impedance bandwidth can be increased, resulting in a decreased radiation efficiency as a cost for this bandwidth increase. The resonance will be split into two close resonances as a result of this slight difference as well.

**Fig 12:**
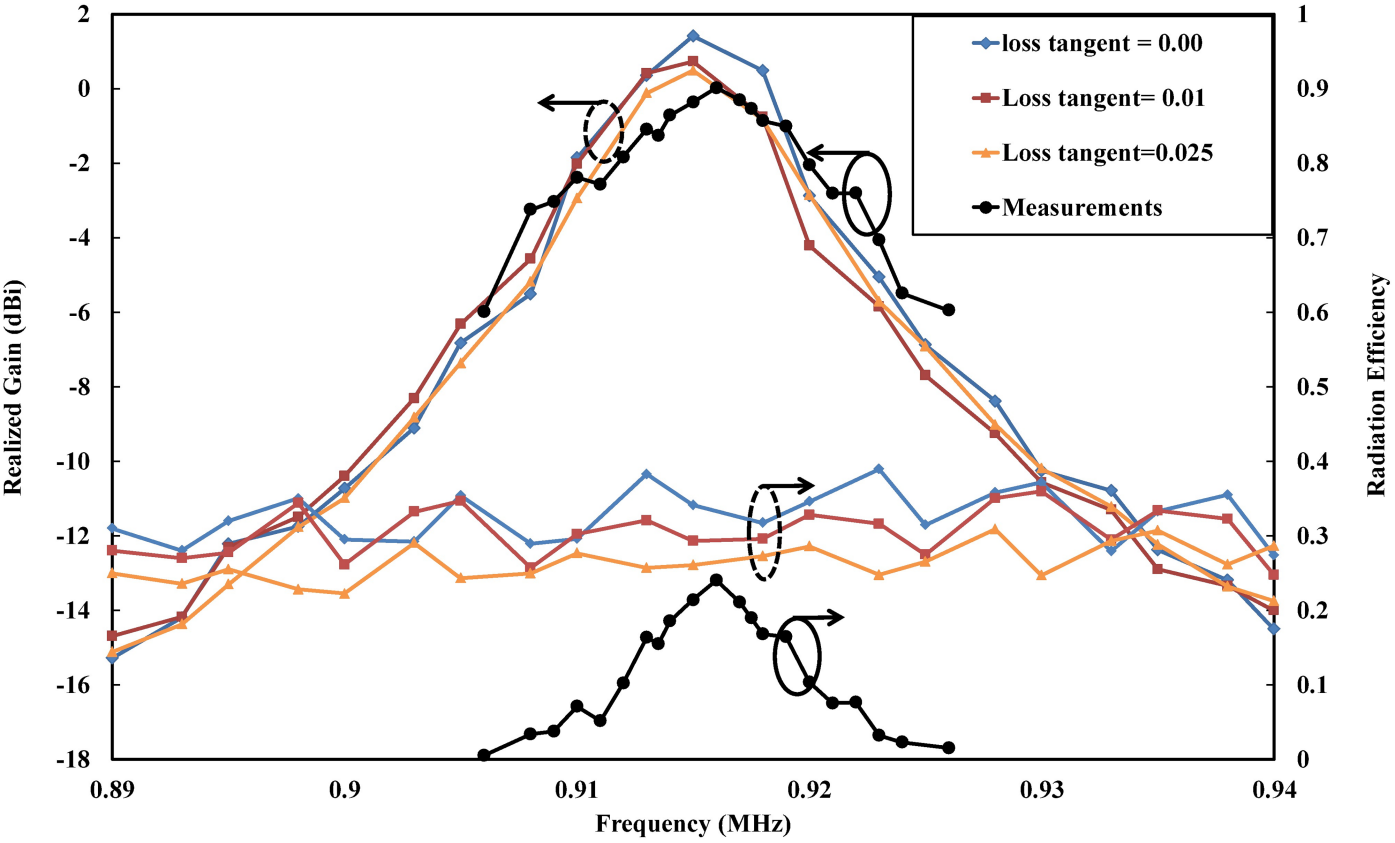
Simulated and measured realized gains and radiation efficiencies of the proposed coplanar tag antenna on a metallic sheet with dimensions of 200 × 200 mm^2^, where the “solid colored lines” represent the simulated values with a loss tangent of an FR4 substrate ranging from 0.00 to 0.025, and the “solid black line” represents the measured values with a loss tangent of 0.025.

Fig 12 illustrates the realized gain and the radiation efficiency of the coplanar designed tag antenna. The simulation results for the realized gain and the range of the efficiency reached 0.48 dBi and 26%–30%, respectively, at a loss tangent equal to 0.025 of the FR4 substrate. Simultaneously, a decrease in the realized gain and radiation efficiency with increased tangent loss can be noticed clearly for the FR4 substrate. In addition, the effect of changing the tangent loss is at the same level for both the realized gain and radiation efficiency, which indicates that improving the matching performance would occur under increasing tangent loss. Meanwhile, we can see some fluctuations in the efficiency curves, which may exist due to the variations in the realized gain.

Furthermore, that enhancement can be noticed well because of the current distribution. The most crucial characteristic for tag antennas is the read range. Most applications of 4-w effective isotropic radiated power (EIRP) [54] need a range of approximately 2.5 m to fulfill the requirement.

We chose metal plates with dimensions of 200 mm × 200 mm, 300 mm × 300 mm, 400 mm × 400 mm, and 500 mm × 500 mm to study the tag performance for metallic applications.

**Fig 13:**
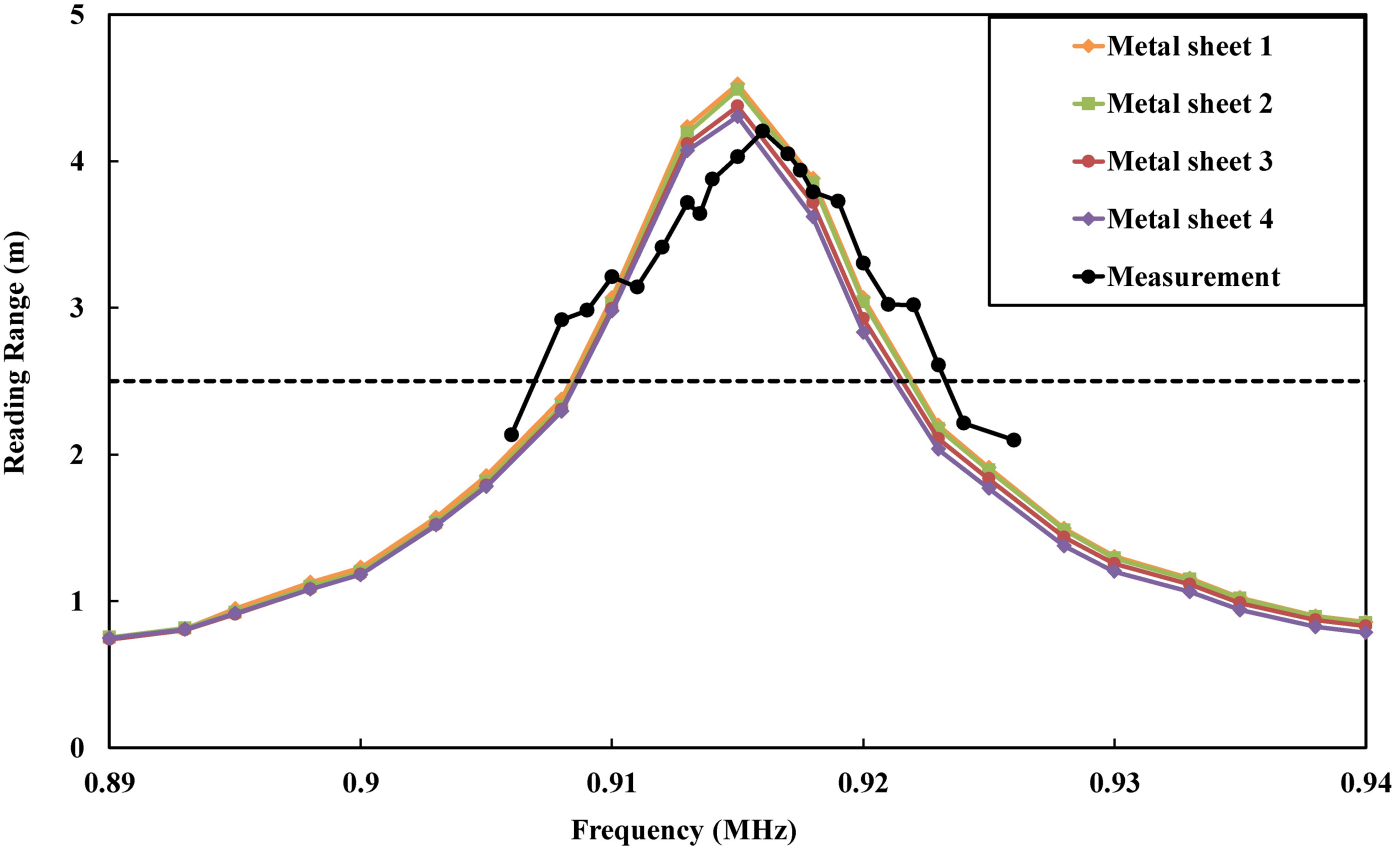
Simulated and measured read ranges of the proposed coplanar tag, where the “solid colored lines” represent the simulated values for the metallic sheet with sizes indicated as Metal sheet 1, Metal sheet 2, Metal sheet 3, and Metal sheet 4, corresponding to 200 × mm 200 mm, 300 × mm 300 mm, 400 mm × 400 mm, and 500 mm × 500 mm. The “solid black line” represents the measured value for the metallic sheet with size indicated by Metal sheet 1.

These metal plates are attached directly to the tag antenna. In all cases, the antenna has a range width of over 2.5 m [2]. Some fluctuations in the read range can be observed when increasing the size of the metal plates; however, this problem will disappear when metal-ground plates are greater than 400 mm × 400 mm. This type of plate is applicable to most metallic objects. For measurement purposes, the minimum tag power for turn on [55] was utilized as a base method. The feasibility and mechanism have been studied in [56] and [57]. The measurement test steps were conducted in an anechoic chamber, where the distance between the tag and the reader was set to 1 m due to the limitation found in the firmware. Measurements were performed under frequencies of (902–928) MHz at a center frequency of 915 MHz. The minimum power for turning on the chip is −7.43 dBm. The realized gain bandwidth at half power bandwidth is 14.5 MHz. The measurements for the realized gain, read range and efficiency are displayed in Figs 12 and 13 separately. From these Figures, we can see that the maximum measured realized gain is 0.01 dBi and that the maximum radiation efficiency is 24% at a maximum reading range of up to 4.2 m. From the results, we can see that the realized gain, read range and radiation efficiency exhibit a good agreement between the simulation and measurement parts, which indicates the good condition of the environment that the measurements were performed under.

It is worthwhile to mention that the chip applied in our proposed design had a power threshold of −8 dBm (160 *μ*W) for MURATA chip and this value is indeed very high if compared to those other types like Higgs for Alien technologies or Texas instrument (e.g. −18 dBm for NXP G2iL, 2015 and Alien Higgs-3, 2012, −20.5 dBm for Alien Higgs-4, 2014, −22.5 dBm for Alien Higgs-EC, 2016). Hence, the tag antenna read range could be improved by threefold [46].

The coplanar antenna was optimized to achieve the maximum realized gain at an acceptable bandwidth. The limitations concerning the tag’s cost and size will decease the radiation efficiency in exchange for improving the impedance matching. In contrast, poor radiation efficiency necessarily results in very low antenna gains. Furthermore, in most miniaturized antennas, making current cancellation difficult to avoid. The enhancement in the efficiency is not outstanding compared to single-layer antennas [58], [59]. The direction of the coplanar antenna is chosen toward improving the reader performance for the coplanar design, where the best method to obtain such results is by increasing the radiation efficiency when a sufficient bandwidth budget is reserved.

## 1 Figure of merit characteristic for electrically small antennas

Many works have been presented Figure of Merit (FoM)for antennas. Recently, Figure of Merit introduced for RFID tag antenna very extensively as a part of study RFID tag performance for ESAs [60].

The first physically small antennas were discussed by Wheeler [61] and Chu [62], who extensively studied the practical and theoretical trade-offs among antenna gain, size, and bandwidth. For a given antenna, the antenna can be called an electrically small antenna when the antenna volume satisfies the requirement *ka* ≤ 0.5, where *k* is the free-space wave number (2*π*/λ) and the radius of an imaginary sphere circumscribing the maximum physical dimension of the antenna. Originally, the quality factor for the most plausible lower bound for a lossy ESA [30], [63] can be calculated from

$$\begin{array}{c} Q_{lb}=\eta *Q_{r}=\eta *\left(\frac{1}{k_{a}}+\frac{1}{n{\left(k_{a}\right)}^{3}}\right). \end{array}$$ (6)

where *n* = 1, 2 for a single-mode and dual-mode (TE or TM) antenna, respectively. Several studies have been conducted to investigate the physical antenna limitations. The ratio of the gain to the quality factor of the maximum value for a directional and omnidirectional ESA was introduced by [54].

$$\begin{array}{c} Q_{dir}^{min}{|}_{small}^{}|\approx \frac{1}{k_{a}}+\frac{1}{2{\left(k_{a}\right)}^{3}},\;\;G_{dir}^{max}{|}_{small}^{}\approx 3. \end{array}$$ (7)

To unequivocally assess the merits of a random electrically small RFID tag antenna, a new Figure of Merit, called normalized bandwidth gain product (NBG), was presented for metallic applications in [60]:

$$\begin{array}{c} NBG=\frac{FBW_{G}\times G_{real}}{k_{d}\times \frac{s-1}{\sqrt{s}}\times \frac{G_{T}}{Q_{lb}}} \end{array}$$ (8)

This infers the closeness of the antenna limitation to its performance. Here, *G*_*real*_ is the maximum realized gain; *FBW*_*G*_ is the realized gain bandwidth; *s* is the maximum allowable gain variation, where a 40% variation in the read range is always set to be at 3-dB; and the quality factor is *kd*. Nevertheless, *d* contains the gap that is extend from the antenna to the metallic surface in addition to the substrate thickness. *G*_*T*_/*Q*_*lb*_ is the ratio of the gain to the quality factor of the maximum possible value for a lossy directional antenna. It is worth mentioning that the value of NBG could be larger than unity; however, larger values are better.

Recently, the performance limits and experiments on ESAs were discussed in [30]. The general bandwidth efficiency *B*_*η*_ product was presented as the FoM.

$$\begin{array}{c} B_{\eta }=\frac{s-1}{\sqrt{s}}\frac{1}{Q_{r}}=\frac{s-1}{\sqrt{s}}*{\left(\frac{1}{k_{a}}+\frac{1}{n{\left(k_{a}\right)}^{3}}\right)}^{-1} \end{array}$$ (9)

[60] has normalized Eq (9) as follows for comparison reasons.

$$\begin{array}{c} NBE=\frac{\sqrt{s}}{s-1}\times FBW_{v}\times \eta \times Q_{r} \end{array}$$ (10)

The value of NBE is always less than one, which is logical considering larger NBE values are preferred.

For each ESA’s mentioned in Table 1, we present the measurement center frequency and its 3-db bandwidth chosen from the *S*_11_ plot or the plot of the antenna gain; both bandwidths are assumed to be the same. To properly compare the variety of bandwidth definitions, the 10-db bandwidth was transformed to a 3-db bandwidth through multiple factors of $2\sqrt{2}$, as concluded in [64]. Various articles have provided realized gain measurements. Although realized gain measurements are very rare, the measurement is more attainable for the read range.

**Table 1:** Characteristics of electrically small tag antennas proposed for metallic applications.

Freq.[MHz]	Size(L×W×H)[mm^3^]	*ε*_*r*_ (tan *δ*)	BW[MHz]	FBW_*G*_[%]	G_*real*_[dBi]	S_11_[dB]	*G*_*tag*_[dBi]	*G*_*T*_[dBi]	*η*[%]	Author [Year]	D[m]	Chip[Ref.]
915	31×19.5×3.065	2.55(0.001)	14.5	1.58	0.01	−15.9	0.12	4.8	24	Proposed Coplanar	4.2^L^	Mu
915	77.68×35.5×1.6^N^	4.6(0.02)	21^c^	2.2	−2.99	−12.5	−2.86	4.8	17.1	Md._2_ [2016]	8.3	G2iL [65]
915	80×44×1.6^N^	4.6(0.02)	290^c^	30.1	−0.09	−24	−0.09	4.8	32	Md._2_ [2015]	11.9	G2iL [66]
923	26×14×2.4	2.65(0.0016)	8	0.87	−1.4	−5.5	0.08	4.8	23	Zhang_2_ [2014]	5.5	NXP [60]
915	36.7×18.1×3.165	4.4(0.02)	38	4.15	−13.9	−23	−13.9	4.8	1.3	Ali [2013]	0.82^L^	Mu [46]
930	28×14×3.2	4.4(0.01)	40	4.3	−11.1	−10	−10.6	4.8	2.8	Zhang_1_ [2013]	1.8^+^	NXP [67]
915	36×36×0.8	4.4(0.02)	91^c^	10.0	−12.8	−19	−12.7	4.8	1.8	Chen^2^ [2010]	1.2	TI [68]
926	32×18×4.2^G^	4.2(0.02)	55	5.9	−11.7	−8.5	−11.0	4.8	2.6	Chen^1^ [2009]	1.5	H2 [69]
911	33.5×20×2	3.0(0.1)	40^c^	4.4	−11.3	−20	−11.3	4.8	2.5	Park [2008]	1.6	H2 [70]
910	42×22×2.2	4.3(0.017)	30	3.3	−4.0	−17	−3.9	4.8	13.5	Son [2008]	NA	H2 [71]
910	34×34×5*	22(0.002)	33	3.6	−4.4	−16	−4.3	4.8	12.4	Kim_2_ [2008]	5.0^L^	H2 [10]
915	25×25×3	48(0.002)	21	2.3	−4.4	−25	−4.4	4.8	12.1	Kim_1_ [2008]	5.0^L^	H2 [21]
911	25×25×3	37(0.002)	13^c^	1.4	−2.8	−17	−2.7	4.8	17.8	Choi_2_ [2008]	6.0^L^	H2 [22]
911	19×19×3	22(0.002)	27^c^	3.0	−7.5	−20	−7.5	4.8	6.0	Choi_1_ [2008]	3.5^L^	H2 [47]

^*G*^ Represents that this antenna size has included the gap from antenna ground plane to the metallic surface;

* represents that this antenna size is given out in form of diameter × diameter × height;

^*c*^ represents that this 3-dB bandwidth is calculated from the corresponding 10-dB one;

^*Mu*, *H*_2_^ and^*T_I_*^ represents that these tag chips are Murata, Higgs-2 and Taxes Instruments respectively;

^*L*^ represents that this read range was measured with a LR reader antenna;

^+^ represents that this read range was measured in frequency band 920–925 MHz (Limitation by firmware).

^*N*^ represents that this antenna is not electrically small antennas (ESA).

$$\begin{array}{c} G_{real}=\tau G_{tag}=\frac{{\left(4\pi \right)}^{2}}{P_{t}G_{t}}\frac{{d_{max}}^{2}}{{\lambda_{o}}^{2}}\frac{Pth}{\rho } \end{array}$$ (11)

Here, λ_*o*_ is the wavelength at the center frequency, *P*_*t*_ *G*_*t*_ is the EIRP, *P*_*th*_ is the tag chip sensitivity. *ρ* Is the mismatch polarization factor, which is set to 1 for LP readers because the tag antenna is mostly linearly polarized (LP), whereas the factor for a circularly polarized reader is 0.5, indicating the presence of a 3-dB polarization mismatch between the antenna and the reader.

As Eq (2) infers from the reflection coefficient of *S*_11_, we can calculate the transmission power *τ* directly; thus, we can calculate the tag gain *G*_*tag*_ from the realized gain *G*_*real*_. The efficiency *η* can also be found according to Eq (12).

$$\begin{array}{c} \eta =\frac{G_{tag}}{G_{T}} \end{array}$$ (12)

*G*_*T*_ is the upper bound of the antenna gain.

As mentioned earlier, this work will follow the above-mentioned steps to obtain results for validation purposes. First, this result will provide us with a clear idea as to the extent of the quality of the tag antenna’s performance in relation to previous works. The most plausible lower bound of the quality factor is *Q*_*lb*_ = 6.28 for single modes. The ratio of the maximum gain to the quality factor as given in Eq (7) is always equal to 3 for directional antennas. Recently, a new FoM concept was introduced for ESAs [60]; therefore, calculating the NBG is very crucial to evaluating the tag antenna performance.

**Fig 14:**
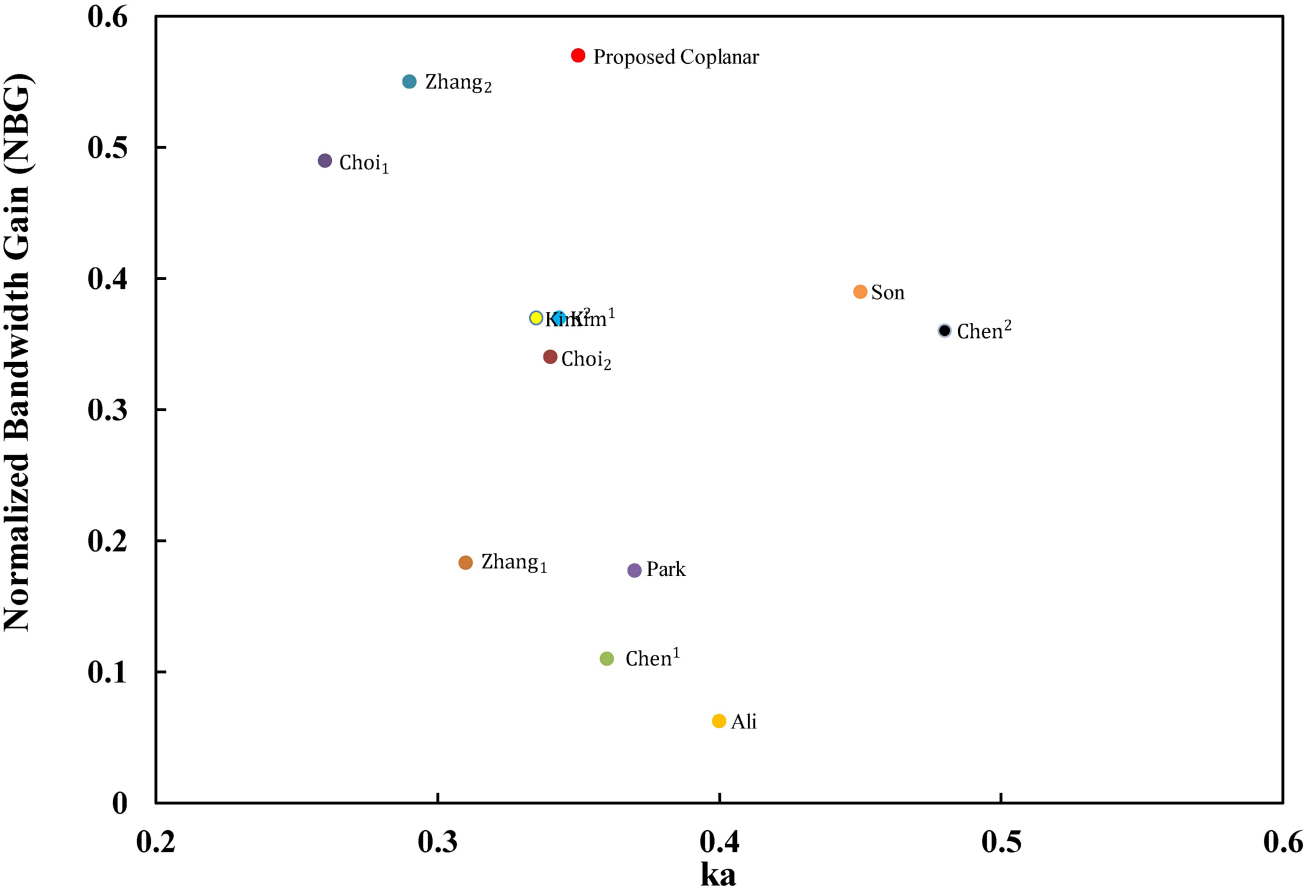
Comparisons of electrically small RFID tag antennas for metallic object applications based on the NBG.

**Fig 15:**
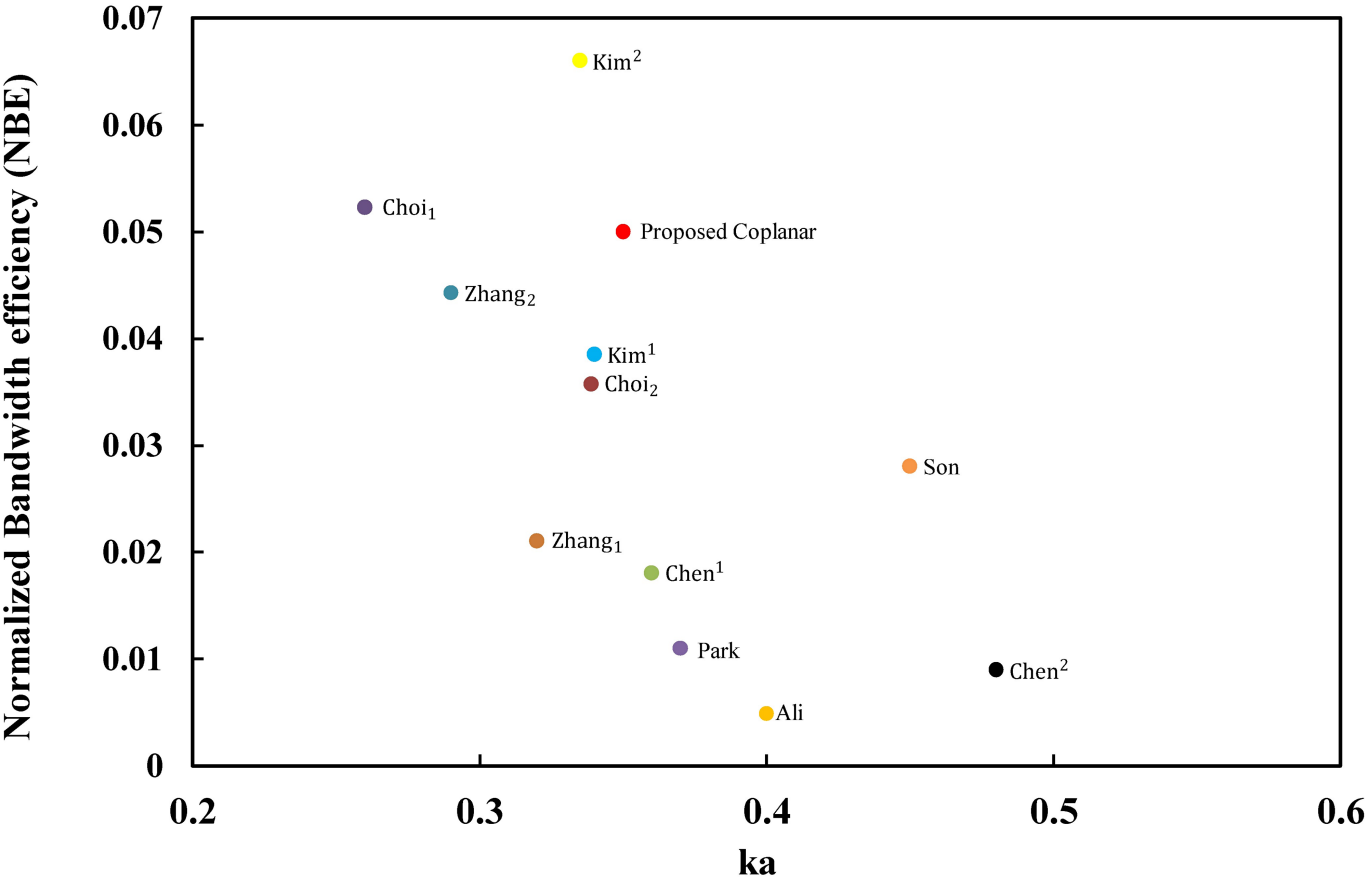
Comparisons of electrically Small RFID tag antennas for metallic object applications based on the NBE.

The results for the coplanar tag design are extracted as mentioned in Table 1. The comparison will be presented here to provide clarity and validation for our design. The overall performance comparisons are performed with both NBG and NBE as the main values in the FoM study. Zhang [60] presented a very clear comparison to validate the ESA, which we can use as part of our comparison. In addition to the comparison illustrated in [60] for the research group at the Electronics and Telecommunications Research Institute (ETRI), we added the tag antenna design that was developed by Ali [46] of Universiti Putra Malaysia for more validation. It’s worth mentioning that we recently added tag antenna designs for Md. Rokunuzzaman [65] and [66] that was improved at RMIT University, Austria to our Table 1 for more validation. Table 1 shows that Md. Rokunuzzaman tag antenna designs are not electrically small antennas where ka for [65] and [66] are 0.84 and 0.88 respectively. Therefore, [65] and [66] will not be included for the overall performance comparisons with both NBG and NBE. The NBG for the proposed coplanar design is better than previous tag designs for metallic objects, as shown in Fig 14, and the NBE results are presented in Fig 15.

## Conclusion

In this paper, we enhanced the gain and achieved bandwidth for electrically small antenna (*ka* ≈ 0.35) RFID tags for metallic applications. This antenna was simulated and found to obtain a good conjugate for the impedance matching part, and the measurement proved this matching. The simulation and measurement proved the reliability of this design with regard to reading performance. This work represents a good candidate for providing a tradeoff between efficiency, bandwidth, and cost. The antenna technique used here is quite unique. The coplanar technology has never been used in this way to improve the radiation efficiency and gain and thus the read range. The gain is equal to 0.12 dBi, and the read range is as high as 4.2 m with a bandwidth-realized gain equal to 14.5 MHz. Compared with works on microstrip, PIFA, conventional meander line and fractal antennas, this coplanar antenna provides greater efficiency and higher gains for equivalent sizes. In this paper, we assessed our tag antenna through a study of the ESA performance. The tag antenna was assessed with a new FoM: the NBG. The NBG plays a role in the design of RFID tag antennas for metallic object applications. The tag antenna equivalent circuit was introduced in this paper for functioning purposes as well as the U-shaped inductive feeder. Furthermore, the tag antenna results are presented based on the NBG and NBE calculations. Finally, a novel coplanar tag antenna RFID for metallic applications was proposed and assessed based on the ESA’s performance.
